# Distribution and molecular characterization of carbapenemase-producing gram-negative bacteria in Henan, China

**DOI:** 10.1038/s41598-024-65106-0

**Published:** 2024-06-22

**Authors:** Chenyu Li, Ruyan Chen, Jie Qiao, Haoyu Ge, Lei Fang, Ruishan Liu, Shuxiu Liu, Qian Wang, Xiaobing Guo, Jianjun Gou

**Affiliations:** 1 Department of Laboratory Medicine, The First Affiliated Hospital of Zhengzhou University, Zhengzhou, China; 2 The Eight Affiliated Hospital of Sun Yat-Sen University, Shenzhen, China; 3 State Key Laboratory for Diagnosis and Treatment of Infectious Diseases, The First Affiliated Hospital, College of Medicine, Zhejiang University, Hangzhou, China; 4 Department of Laboratory Medicine, The Fifth Affiliated Hospital of Zhengzhou University, Zhengzhou, China; 5 Department of Laboratory Medicine, The First Affiliated Hospital of Henan University of Chinese Medicine, Zhengzhou, China

**Keywords:** Carbapenem-resistant gram-negative bacteria, Carbapenemase-producing gram-negative bacteria, Multidrug-resistant, KPC, NDM, Antimicrobials, Environmental microbiology, Policy and public health in microbiology

## Abstract

This study aimed to investigate the epidemiological characteristics and trends over time of carbapenemase-producing (e.g., KPC, NDM, VIM, IMP, and OXA-48) Gram-negative bacteria (CPGNB). Non-duplicated multi-drug resistant Gram-negative bacteria (MDRGNB) were collected from the First Affiliated Hospital of Zhengzhou University from April 2019 to February 2023. Species identification of each isolate was performed using the Vitek2 system and confirmed by matrix-assisted laser desorption ionization-time of flight mass spectrometry according to the manufacturer's instructions. PCR detected carbapenem resistance genes in the strains, strains carrying carbapenem resistance genes were categorized as CPGNB strains after validation by carbapenem inactivation assay. A total of 5705 non-repetitive MDRGNB isolates belonging to 78 different species were collected during the study period, of which 1918 CPGNB were validated, with the respiratory tract being the primary source of specimens. Epidemiologic statistics showed a significant predominance of ICU-sourced strains compared to other departments. *Klebsiella pneumoniae*, *Escherichia coli*, *Acinetobacter baumannii*, and *Pseudomonas aeruginosa* were the significant CPGNB in Henan, and KPC and NDM were the predominant carbapenemases. Carbapenem-resistant infections in Henan Province showed an overall increasing trend, and the carriage of carbapenemase genes by CPGNB has become increasingly prevalent and complicated. The growing prevalence of CPGNB in the post-pandemic era poses a significant challenge to public safety.

## Introduction

Carbapenem antibiotics are a class of β-lactam antibiotics with a broad antimicrobial spectrum, vigorous antimicrobial activity, stability to β-lactamases, and low toxicity. They are regarded as one of the most critical antibiotics for multidrug-resistant gram-negative bacteria^1^. However, in recent years, carbapenem-resistant gram-negative bacteria (CRGNB) have shown a significant upward trend with the clinical use of carbapenem antibiotics^2–4^. Mechanisms of resistance in CRGNB can be divided into three side: (1) production of carbapenemases, (2) absence or alteration of outer membrane pore proteins, and (3) overexpression of efflux pumps^5^. One of the most prominent pathways is the production of carbapenemases. Ambler's classification suggests carbapenemases are mainly categorized as A, B, and D^6^. Category A is a serine enzyme, such as KPC, which is most commonly found in *Klebsiella pneumoniae* but also in *Pseudomonas aeruginosa* and *Acinetobacter baumannii*; category B carbapenemases, also known as metallo-beta-lactamases (MBLs)^7^, which are chromosome-mediated, such as NDM, IMP, and VIM; and category D carbapenemases are serine enzymes such as OXA, which are commonly found in *K. pneumoniae* and *A. baumannii*.

In 2017, WHO classified CRGNB as a serious societal security threat, which mainly includes carbapenem-resistant Enterobacteriaceae (CRE) and carbapenem-resistant non-fermenting Gram-negative bacteria (CRNFGNB) (mainly *P. aeruginosa* and *A. baumannii*). CRGNB infections often occurs in patients with severe underlying diseases, immunodeficiencies, and long-term antibiotic use, and the prognosis for patients with infections is poor due to the limited availability of medications. The mortality rate of patients with CRGNB infections ranges from 27 to 44%^8^. Infections caused by CRGNB have become a global problem^6,9^.

Although the attention to carbapenem-resistant strains has increased at home and abroad in recent years^10^, epidemiologic data about the Henan region are still deficient. This study focuses on the statistical analysis of the epidemiology of CRE in Henan in the past four years.

## Material and methods

### Collection of specimens

MDGNB were collected between April 2019 and February 2023 from the Laboratory Department of the First Affiliated Hospital of Zhengzhou University. Identical strains from the same specimen source from the same patient within three months were defined as duplicate strains and excluded. Isolated strains were stored at − 80 °C.

### Strain identification

Specimens were used to collect specimen information corresponding to patient hospitalization number, name, gender, age, department, specimen number, specimen date, specimen type, and strain information through a laboratory information management system. Species identification of each isolate was performed using the Vitek2 system and confirmed by matrix-assisted laser desorption ionization-time of flight mass spectrometry according to the manufacturer's instructions (*Escherichia coli* ATCC8739 was used as the quality control strain), and the VITEK-compact 2 automatic microbial system was used for the antimicrobial susceptibility test (AST) to determine multi-drug resistance of the strains.

### Screening and characterization of clinically significant carbapenemases

Polymerase chain reaction was used to detect carbapenem resistance genes of strains, including *bla*_KPC_, *bla*_NDM_, *bla*_IMP_, *bla*_VIM_, and *bla*_OXA-48_*.* Strains carrying carbapenem resistance genes were categorized as carbapenemase-producing strains after validation of carbapenem inactivation assay.

### Statistical analysis

Differences in carbapenem-resistant strains across epidemiologic characteristics and trends over time were assessed using a chi-square test with an overall significance level of 5%.

## Results

### Overview of multidrug-resistant Gram-negative *bacteria* isolates

From April 2019 to February 2023, a total of 5705 non-repetitive MDRGNB isolates belonging to 78 different strains were collected from the Laboratory Department of the First Affiliated Hospital of Zhengzhou University, screened for carbapenem genes by PCR and verified by carbapenem inactivation experiments. The results showed a total of 1918 CPGNB, including 1339 (69.81%) *K. pneumoniae*, 164 (8.55%) *E. coli*, 140 (7.30%) *A. baumannii*, 94 (4.90%) *P. aeruginosa*, 43 (2.24%) *Enterobacter cloacae* (ECC) and 138 (7.19%) uncommon strains, including *Morganella morganii*, *Proteus mirabilis*, *Enterobacter aerogenes*, *Citrobacter freundii*, *Klebsiella oxytoca*, *Providencia rettgeri*, *Serratia marcescens* and so on. Among them, 1501 (78.26%) isolates carried *bla*_KPC_, 418 (21.79%) isolates carried *bla*_NDM_, 54 (2.82%) isolates carried *bla*_IMP_, 35 (1.82%) isolates carried *bla*_VIM_, and 17 (0.89%) isolates carried *bla*_OXA-48_. The above data indicated that *K. pneumoniae*, *E. coli*, *A. baumannii*, and *P. aeruginosa* were the significant CPGNB in Henan, and KPC and NDM were the major carbapenemases among the prevalent multi-drug resistant strains in Henan. Distribution of the number of different carbapenemase species in CPGNB year-by-year is shown in Supplementary Table S1.

### Epidemiological characteristics and annual trend of CPGNB

**Figure 1:**
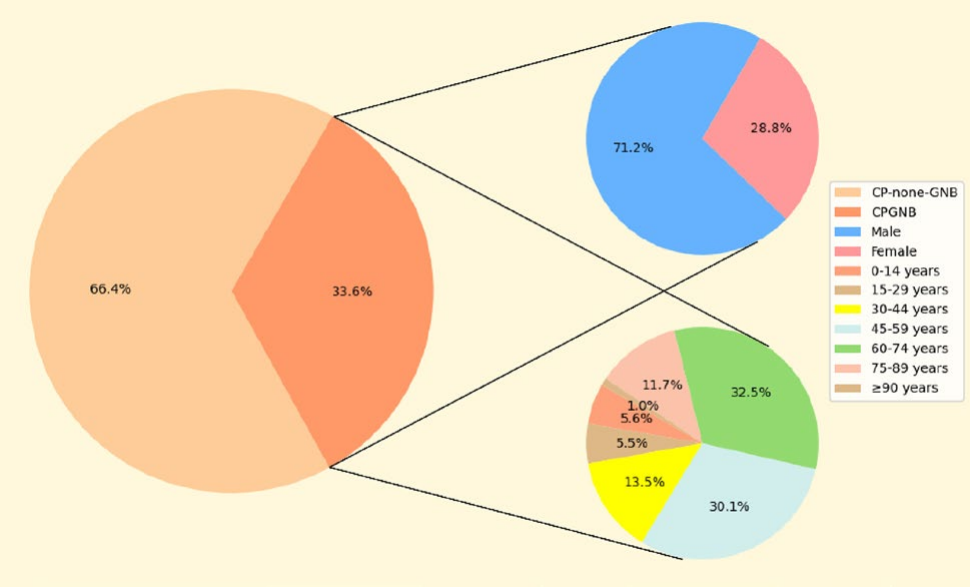
The percentage of CPGNB in the MDR strains collected in this study and the gender and age distribution among them.

The chi-square test assessed trends in the epidemiologic characteristics of CPGNB isolates over the study period. As shown in Fig. 1, of the 1918 CPGNB isolates collected in this study from 2019 to 2023, 1365 were sourced from male and 553 from female patients. The prevalence of CPGNB infection in males was significantly higher than in females (P < 0.0001). This distributional characteristic is consistent with the distribution of patients from the MDR sample source, so the statistical results of the present study may be a generalized phenomenon due to the variability of the clinically ill persons themselves, as also presented in the study of Jinnethe Reyes^4^.

Except for 2020, the proportion of CPGNB in total MDR isolates increased year by year, from 32.71% in 2019 to 34.08% in 2021, 36.00% in 2022, and 45.64% in 2023, while CPGNB accounted for only 22.24% in 2020, which may be influenced by the COVID-19 pandemic, when, in an attempt to control the epidemiology of the COVID-19 pandemic, China adopted a series of control measures and organized additional square-cabin hospitals to treat febrile and infected patients, and the number of general hospital admissions dropped significantly. In terms of age, CPGNB infections were concentrated in middle-aged and elderly patients (median age 59), with patients aged 45–74 years being the primary source of specimens from 2019 to 2023, and it is noteworthy that the rate of CPGNB infections in patients aged 60 years or older increased in 2023. At the end of 2022, China liberalized its control of neocoronary pneumonia. The number of COVID-19 infections improved significantly, with those 60 Patients over 60 years of age more susceptible to CPGNB attacks due to weakened resistance or underlying diseases such as diabetes and hypertension, which may have contributed to the high percentage of patients over 60 years of age infected with CPGNB in 2023. According to the proportion of different species isolated in other years, it can be observed that, as mentioned earlier, *K. pneumoniae* is the primary strain of CPGNB in Henan. As a conditionally pathogenic bacterium, *K. pneumoniae* mainly colonizes the upper respiratory tract and the intestinal tract of the human body and enters the lungs through the respiratory tract to cause substantial lesions when the body's resistance is lowered. *K. pneumoniae* is considered the most common cause of nosocomial pneumonia. It accounts for 3 to 8% of all nosocomial bacterial infections^11^. The percentage of *K. pneumoniae* showed a significant decreasing trend (P < 0.05) during the strict control of the novel coronavirus epidemic from 2020 to 2022. This lower rate may be mainly related to increased awareness of wearing masks and paying attention to hand hygiene during the COVID-19 pandemic, and significantly increased (P < 0.05) after the liberalization of control measures in 2023, which may imply a specific correlation between novel coronavirus pneumonia and *K. pneumoniae* infection.

### High prevalence of CPGNB in the ICU department

**Figure 2:**
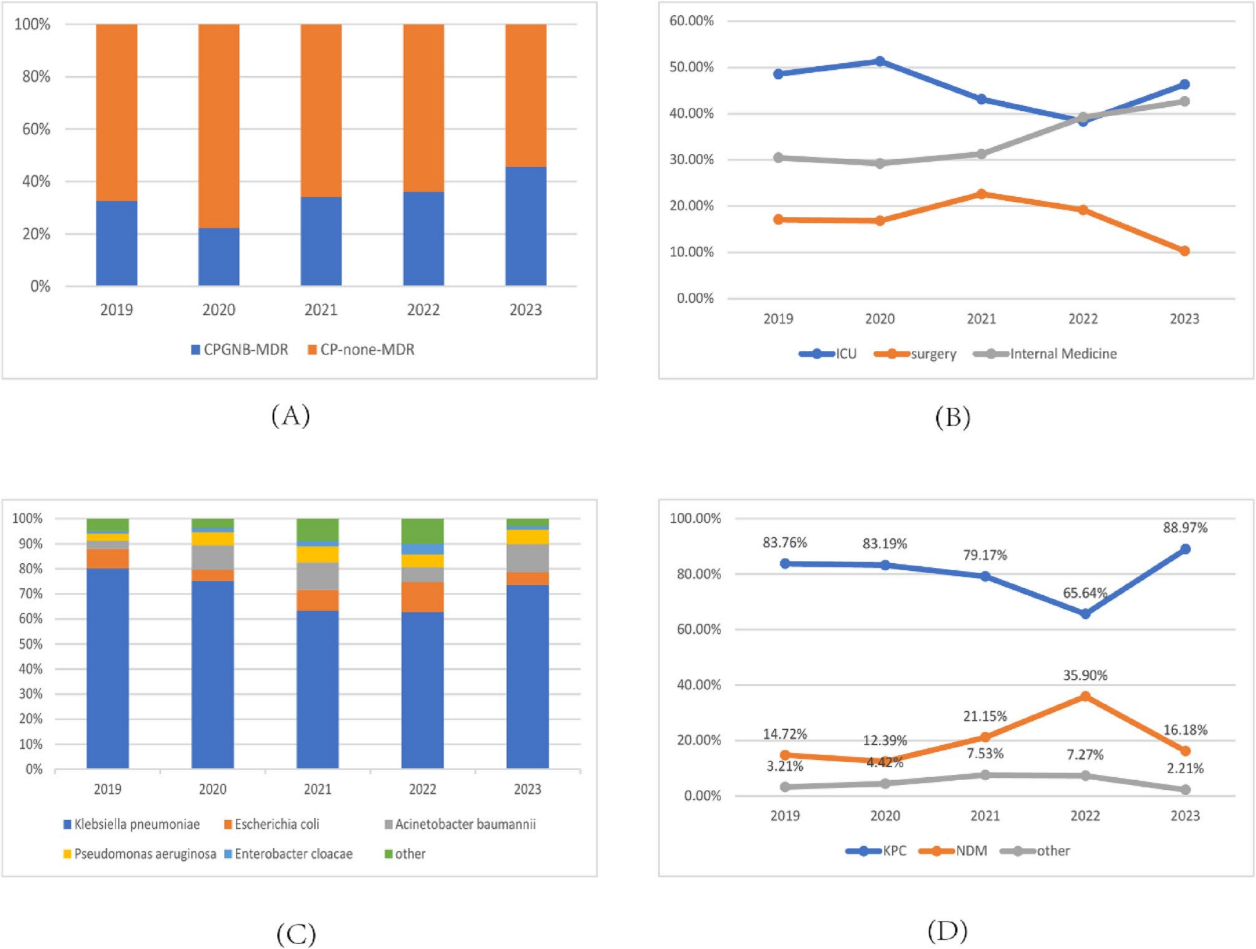
(**A**) Trends in CPGNB as a percentage of MDR over the study period, (**B**) distribution of CPGNB infections in different departments and changes in trend, (**C**) distribution of different species strains in CPGNB and changes in trend, (**D**) expression of different species of carbapenemases in CPGNB and changes in trend.

As shown in the Fig. 2, among the 1918 CPGNB strains, ICU-derived strains (851/1918) had a significant advantage over other departments, such as internal medicine (644/1918) and surgery (362/1918). From 2019 to 2023, the percentage of ICU-sourced CPGNB in the total CPGNB of that year was 48.56%, 51.33%, 43.11%, 38.33%, and 46.32%, respectively. In the aftermath of the COVID-19 outbreak, the ICU isolation rate was significantly lower in 2022 than in 2020 and then considerably higher in 2023. Among the 851 CPGNB isolates from ICUs, carbapenemase-producing *Klebsiella pneumoniae* (CP-KP) accounted for the most significant proportion (679/851), much higher than *A. baumannii* (68/851), *P. aeruginosa* (38/851), and *E. coli* (29/851). In addition to this, it is worth noting that except for 2020, the percentage of CPGNB from internal medicine sources is in an increasing trend, from 30.46% in 2019 to 31.25% in 2021, to 39.21% in 2022 and 42.65% in 2023, where it even surpasses the ICU as the departmental division with the highest percentage in 2022, which may portend the growing drug resistance potential in internal medicine.

### Potentially high CPGNB infection rates for bloodstream infections

The 1918 CPGNB included 1072 (55.89%) specimens of respiratory origin, 196 (10.22%) specimens of blood origin, 187 (9.75%) specimens of urinary origin, 182 (9.49%) specimens of other sterile body fluid origin, and 281 (14.65%) specimens of other bacterial body fluid origin, with respiratory, blood and urine being the major specimen sources. In addition, the ratio of CPGNB to MDR isolated from specimens of respiratory, blood and urine sources were 34.30% (1072/3125), 38.58% (196/508), 22.08% (187/847) respectively. There was a significantly higher frequency of CPGNB isolation in MDR that triggered bloodstream infections(BSI) compared to the others.

### The prevalence of Carbapenem enzyme gene and the trend of increasing complexity

As mentioned above, in recent years, carbapenemase-producing strains have generally shown an increasing trend. We plotted the year-to-year trend of the percentage of different carbapenemase-producing strains in the CPGNB in the time-year (Fig. 2). It is evident that KPC is always the most common type of carbapenemase and, as in the case of *K. pneumoniae* from 2019 to 2023, the KPC enzyme-producing strains were significantly decreased (P < 0.01) from 2019 to 2023 and then considerably rebounded (P < 0.01) in the 2023 year significantly reversed (P < 0.01). Among the KPC-producing CPGNB, the most common was *K. pneumoniae* (1289/1501), followed by *A. baumannii* (89/1501), *P. aeruginosa* (42/1501), *E. coli* (28/1501), etc. In addition to this, NDM was also the main prevalent carbapenemase in Henan. Compared with the very high percentage of *K. pneumoniae* in the KPC-producing CPGNB, the distribution of strains in the NDM-producing CPGNB showed a more homogeneous character, in which the most common NDM-producing was *E. coli* (138/418), followed by *K. pneumoniae* (83/418), *A. baumannii* (48/418), and ECC (39/418), *P. aeruginosa* (26/418) among others. In addition to the significant carbapenemases, we tested three carbapenemase genes: IMP, VIM, and OXA-48. *P. aeruginosa* (31/54) was the most common IMP-producing CPGNB, followed by *K. pneumoniae* (10/54), and IMP expression was also seen in *A. baumannii* (2/54) and *E. coli* (2/54). VIM was mainly expressed by *Pseudomonas spp.* (15/35), such as *P. aeruginosa*, *P. falciparum*, and *P. scherzingeri*. CPGNB expressing OXA-48 were less common, of which the primary strain was *Klebsiella* spp. (9/17), including six strains of *K. pneumoniae*, two strains of *Klebsiella aerogenes*, and one strain of *Klebsiella metapneumoniae*, in addition to *A. baumannii* (6/17), which was also seen to express OXA-48.

It is worth mentioning that, in addition to the widespread of carbapenem-resistant antibiotic genes, the complex trend of a single strain expressing two or more carbapenemases simultaneously is worrisome. The Table 1 shows the number of strains expressing two or more carbapenemases per year during this study. GNB expressing multiple carbapenemases were detected in every year except 2020. There was a significant increase in GNB representing a total of two carbapenemases in 2021 (6.89%) compared to 2019 (1.69%) (P < 0.0001), followed by 2022 (8.37%) and 2023 (7.35%), which also maintained a high level. The strains expressed mostly dual carbapenemases, KPC-NDM, the two most common carbapenemases.

**Table 1:** Distribution of the number of strains co-expressing multiple carbapenemases in CPGNB during the study period.

		2019	2020	2021	2022	2023
Number of strains co-expressing two carbapenemase	KPC + NDM	7		26	25	8
	KPC + IMP	2		6	7	1
	KPC + VIM			7	1	
	KPC + OXA-48			1		
	NDM + IMP	1			2	1
	NDM + VIM			3	2	
	NDM + OXA-48				1	
Number of strains co-expressing three carbapenemase	KPC + NDM + IMP			1	1	

In addition to this, two CPGNB strains carrying more than two carbapenemase genes at the same time were collected during this study, isolated from 2021 and 2022, respectively. Both strains were *C. freundii* carrying both *bla*_KPC_, *bla*_IMP_ and *bla*_NDM_, and the two strains were isolated from different departments, patients, and different types of body fluids. These two non-repetitive strains of *C. freundii* are resistant to most antibiotics, and only sensitive to polymyxin B, amikacin, and fosfomycin. Isolated from *C. freundii* from 2021 intermediate to tigecycline, another strain isolated from 2022 intermediate to tigecycline, levofloxacin and trimethoprim-sulfamethoxazole. Although the number of GNB co-expressing multiple carbapenemases has not increased dramatically, the potential pitfalls of such GNB co-expressing complex carbapenemases are also significant.

## Discussion

During the four-year surveillance period from April 2019 to March 2023 of this study, the percentage of CPGNB in clinical MDR in Henan increased significantly, while the considerably lower rate presented in 2020 was most likely affected by the COVID-19 epidemic. To curb the spread of the COVID-19 epidemic, China has adopted a series of measures to control the movement of people and the policy of isolating and treating people infected with the COVID-19 and has achieved good results. On the one hand, the activity of people in society has been dramatically decreased, and the number of outpatient visits to general hospitals and the volume of clinical specimens has significantly been reduced. Secondly, hospitals adopted more stringent isolation and sterilization policies during the COVID-19 epidemic to increase the awareness of healthcare workers (HCWs) to comply with infection control measures and hand hygiene, significantly reducing the likelihood of pathogenic microorganisms such as COVID-19 and CPGNB transmission. Olivier Lemenand's study also observed a decrease in the prevalence of French-produced ESBL Enterobacteriaceae in 2020^12^. At the same time, the speculations of Clarisse Duverger et al. agree with the present study^13^.

However, despite a transient reduction in CPGNB prevalence in 2020, overall, CPGNB prevalence still shows a rising trend, especially for CRE caused by carbapenem-resistant *Klebsiella pneumoniae* (CRKP). Rapid increases in the prevalence of CRE have been reported globally^14–16^. In contrast, *K. pneumoniae* accounts for the country's most significant proportion of CPGNB strains and is the species with the fastest-growing infection rate^17^. Meanwhile, the data of this study showed that *K. pneumoniae* was the most predominant CPGNB in Henan Province, which was consistent with the results of previous studies.^18^ The China Antimicrobial Resistance Surveillance Report (http://www.carss.cn/), the largest survey of antimicrobial resistance in China, reported that in recent years, the resistance rate of *K. pneumoniae* to carbapenems in Henan has been much higher than the national average. According to studies, ST11 is the most common subtype of *K. pneumoniae*^19,20^, and in recent years, the frequency of carbapenem-resistant *hypervirulent Klebsiella pneumoniae* (CR-hvKP) production has increased significantly, especially in KPC-2-producing ST11. Dissemination of hypervirulence could be extremely rapid due to limited fitness costs^21^. This suggests that CP-KP is the biggest concern of CPGNB in Henan Province.

Bloodstream infection (BSI) is a serious complication that often occurs in the ICU and is often associated with high mortality in infected patients. Whereas BSI due to CPGNB is more severe, D Ben-David's study showed that infection-related mortality was significantly higher among patients with CRKP, compared with those with extended-spectrum β-lactamase-producing *Klebsiella pneumoniae* (ESBLKP) or susceptible *Klebsiella pneumoniae* (SKP) BSI (48%, 22% and 17%, respectively)^22^. The results of the present study also showed that blood-derived CPGNB accounted for a higher percentage of its corresponding MDR compared to other body fluid sources, which may suggest that CPGNB, while possessing greater drug resistance, is also more likely to cause serious complications in infected patients and thus lead to a high mortality rate.

Previous studies have demonstrated that production of carbapenemases is the primary mechanism of carbapenem antibiotic resistance in Chinese CRE strains, and the most prominent carbapenemases are KPC and NDM^15,23,24^, which were reconfirmed in this study. In addition, the data in this study indicate that in recent years, CPGNB carrying rare carbapenemase genes (*bla*_OXA-48_, *bla*_IMP_, *bla*_VIM_), etc., have been gradually increasing as CPGNB gradually spread and evolved.

At the same time, the evolution of CPGNB from a single isolate carrying a total of two or more carbapenemase genes is increasing, and the prevalence of carbapenemase genes among strains is becoming increasingly complex. In this study, two non-replicated strains were collected that simultaneously carried *bla*_KPC_, *bla*_IMP_ and *bla*_NDM_ of *C. freundi*. Qiao J performed a detailed sequencing analysis of *C. freundii* carrying *bla*_KPC-2_, *bla*_IMP-4_, *bla*_*OXA-1*_, and *bla*_NDM-1_ isolated from 2021^25^. The *bla*_IMP-4_ coexists with the *bla*_NDM-1_ on a novel transferable plasmid, while the *bla*_KPC-2_ is located on another plasmid stably coexisting in this bacterial strain. This result suggests that as plasmids carrying carbapenemase genes continue to evolve, not only can a single plasmid carry multiple carbapenemase genes at the same time, but multiple plasmids carrying resistance genes can also stably coexist in a single strain of drug-resistant bacteria, thus generating a pan-drug-resistant CPGNB, which may pose a huge problem in clinical treatment.

It is worth noting that COVID-19 hospitalized patients at Intensive Care Units (ICU) share underlying diseases associated and risk factors for bacterial and fungal infections, such as corticosteroid therapy, chronic respiratory diseases, intubation/mechanical ventilation, and immunoinflammatory response (cytokine storm)^26^. In the process, as the patient's microbiota is exposed to such complex environments, the carbapenemase gene is more likely to evolve within the host and the likelihood of transmission between hosts is further increased. At the same time, there may be an increment in the severity of Healthcare-Associated Infections resulting from the exposure of the patient's microbiota to these factors through the selection, emergence, and spread of resistance factors and more virulent microorganisms. The significantly higher CPGNB infection rate in 2023 in this study suggests that the spread and evolution of the carbapenemase gene may be further accelerated and complicated by the ongoing liberalization of controls for new crown pneumonia^27,28^. This could significantly challenge public health and effective infection control.

## Ethical approval

This study was approved by the Ethics Committee of The first affiliated hospital of Zhengzhou University, Henan, China (2023-KY-1109). The First Affiliated Hospital of Zhengzhou University Ethics Committee also approved the waiver of informed consent to participate in this study owing to its retrospective design. All patient data were anonymised prior to analysis. Study procedures were conducted in accordance with the ethical standards of the Declaration of Helsinki.

### Supplementary Information

Supplementary Information.

## Data Availability

The datasets generated and/or analysed during the current study are not publicly available due to the protection of personal privacy but are available from the corresponding author on reasonable request.
