# Supplementary material for: Distribution and molecular characterization of carbapenemase-producing gram-negative bacteria in Henan, China
Source: Sci Rep. 2024 Jun 22;14:14418. doi: 10.1038/s41598-024-65106-0 (PMC11193736; doi:10.1038/s41598-024-65106-0)
Supplement: Supplementary file 1 — Supplementary Information. [file 41598_2024_65106_MOESM1_ESM.docx]

|  |  | 2019 | 2020 | 2021 | 2022 | 2023 |
| --- | --- | --- | --- | --- | --- | --- |
| *Klebsiella pneumoniae* | KPC | 463 | 84 | 380 | 261 | 100 |
|  | NDM | 15 | 1 | 27 | 34 | 6 |
|  | IMP | 1 | 0 | 2 | 7 | 0 |
|  | VIM | 0 | 0 | 4 | 0 | 0 |
|  | OXA | 0 | 0 | 2 | 4 | 0 |
| *Escherichia coli* | KPC | 9 | 2 | 12 | 4 | 1 |
|  | NDM | 38 | 3 | 38 | 52 | 7 |
|  | IMP | 0 | 0 | 2 | 0 | 0 |
|  | VIM | 0 | 0 | 2 | 0 | 0 |
|  | OXA | 0 | 0 | 1 | 0 | 0 |
| *Acinetobacter baumannii* | KPC | 13 | 7 | 48 | 9 | 12 |
|  | NDM | 6 | 1 | 22 | 15 | 4 |
|  | IMP | 1 | 0 | 0 | 1 | 0 |
|  | VIM | 0 | 0 | 0 | 2 | 0 |
|  | OXA | 1 | 3 | 2 | 0 | 0 |
| *Pseudomonas aeruginosa* | KPC | 5 | 0 | 24 | 7 | 7 |
|  | NDM | 6 | 4 | 8 | 9 | 0 |
|  | IMP | 7 | 2 | 12 | 8 | 2 |
|  | VIM | 0 | 0 | 3 | 1 | 0 |
|  | OXA | 0 | 0 | 0 | 1 | 0 |
| *Enterobacter cloacae* | KPC | 0 | 0 | 3 | 2 | 0 |
|  | NDM | 6 | 2 | 10 | 19 | 2 |
|  | IMP | 0 | 0 | 1 | 1 | 0 |
|  | VIM | 0 | 0 | 1 | 0 | 0 |
|  | OXA | 0 | 0 | 0 | 0 | 0 |
| others | KPC | 5 | 1 | 27 | 15 | 1 |
|  | NDM | 16 | 3 | 27 | 34 | 3 |
|  | IMP | 1 | 0 | 2 | 2 | 1 |
|  | VIM | 8 | 0 | 12 | 4 | 0 |
|  | OXA | 0 | 0 | 1 | 2 | 0 |

Table S1 Year-by-year distribution of the number of different carbapenemase species in carbapenemase-producing Gram-negative bacilli.
